# Supplementary material for: An open-source framework for large-scale, flexible evaluation of biomedical text mining systems
Source: J Biomed Discov Collab. 2008 Jan 29;3:1. doi: 10.1186/1747-5333-3-1 (PMC2276192; doi:10.1186/1747-5333-3-1)
Supplement: Additional file 1 — Evaluation platform source code. The additional file contains the software evaluation framework discussed in this paper. The most current release can be downloaded from SourceForge.net [1]. See the accompanying README file for instructions on installation and use. Also included in the distribution is a collection of UIMA wrappers for some commonly used BioNLP tools and annotated corpora. [file 1747-5333-3-1-S1.zip › bionlp_uima_repository-1.0.1/docs/api/src-html/edu/uchsc/ccp/uima/ae/util/filter/OverlappingAnnotationResolver_AE.html]

```
001    package edu.uchsc.ccp.uima.ae.util.filter;
002    
003    import java.util.ArrayList;
004    import java.util.Arrays;
005    import java.util.HashMap;
006    import java.util.HashSet;
007    import java.util.Iterator;
008    import java.util.List;
009    import java.util.Map;
010    import java.util.Set;
011    
012    import org.apache.uima.analysis_engine.ResultSpecification;
013    import org.apache.uima.analysis_engine.annotator.AnnotatorConfigurationException;
014    import org.apache.uima.analysis_engine.annotator.AnnotatorContext;
015    import org.apache.uima.analysis_engine.annotator.AnnotatorContextException;
016    import org.apache.uima.analysis_engine.annotator.AnnotatorInitializationException;
017    import org.apache.uima.analysis_engine.annotator.AnnotatorProcessException;
018    import org.apache.uima.analysis_engine.annotator.JTextAnnotator_ImplBase;
019    import org.apache.uima.cas.CASException;
020    import org.apache.uima.jcas.JCas;
021    import org.apache.uima.jcas.cas.FSArray;
022    
023    import edu.uchsc.ccp.uima.annotation.CCPAnnotationSet;
024    import edu.uchsc.ccp.uima.annotation.CCPDocumentInformation;
025    import edu.uchsc.ccp.uima.annotation.CCPTextAnnotation;
026    import edu.uchsc.ccp.uima.util.UIMA_Util;
027    import edu.uchsc.ccp.util.nlp.annotation.InvalidSpanException;
028    import edu.uchsc.ccp.util.nlp.annotation.Span;
029    
030    /**
031     * Default behavior is to keep the longest annotation for any that overlap. The user also has the option to keep the longest common substring among
032     * all overlapping annotations.
033     * 
034     * <br>
035     * <br>
036     * Known bug in longest common substring:<br>
037     * If you have three overlapping annotations<br>
038     * <nbsp><nbsp>11111111<br>
039     * <nbsp><nbsp><nbsp>222222222222222222<br>
040     * <nbsp><nbsp><nbsp><nbsp>333333333333333333333333<br>
041     * <br>
042     * It is possible that the code will compare annotations 1 and 2 first, and result in a longest common substring of 22, and then miss completely the
043     * longest common substring between 2 and 3. <br>
044     * <br>
045     * LONGEST COMMON SUBSTRING FEATURE DOES NOT CURRENTLY WORK
046     * 
047     * @author williamb
048     * 
049     */
050    public class OverlappingAnnotationResolver_AE extends JTextAnnotator_ImplBase {
051    
052        private  boolean DEBUG = false;
053    
054        public final String PARAM_ANNOTATION_SETS_TO_IGNORE = "AnnotationSetsToIgnore";
055    
056        public final String PARAM_KEEP_LONGEST_COMMON_SUBSTRING = "KeepLongestCommonSubstring";
057    
058        /**
059         * The annotation type to resolve overlaps
060         */
061        public final String PARAM_ANNOTATION_TYPE_OF_INTEREST = "AnnotationTypeOfInterest";
062    
063        protected Set<Integer> annotationSetsToIgnore;
064    
065        protected String annotationTypeOfInterest;
066    
067        protected boolean keepLongestCommonSubstring;
068    
069        @Override
070        public void initialize(AnnotatorContext ac) throws AnnotatorInitializationException, AnnotatorConfigurationException {
071    
072            try {
073                annotationTypeOfInterest = (String) ac.getConfigParameterValue(PARAM_ANNOTATION_TYPE_OF_INTEREST);
074                keepLongestCommonSubstring = (Boolean) ac.getConfigParameterValue(PARAM_KEEP_LONGEST_COMMON_SUBSTRING);
075                try {
076                annotationSetsToIgnore = new HashSet<Integer>(Arrays.asList((Integer[]) ac
077                        .getConfigParameterValue(PARAM_ANNOTATION_SETS_TO_IGNORE)));
078                } catch (NullPointerException npe) {
079                    annotationSetsToIgnore = new HashSet<Integer>();
080                }
081            } catch (AnnotatorContextException e) {
082                e.printStackTrace();
083            }
084    
085            super.initialize(ac);
086        }
087    
088        /**
089         * Default behavior is to keep the longest of any overlapping annotation
090         */
091        public void process(JCas jcas, ResultSpecification arg1) throws AnnotatorProcessException {
092            /* cycle through each protein mention... if it overlaps with another protein mention, remove the shorter one? */
093    
094            /* get document ID */
095            Iterator docInfoIter = jcas.getJFSIndexRepository().getAnnotationIndex(CCPDocumentInformation.type).iterator();
096            String documentID = "-1";
097            if (docInfoIter.hasNext()) {
098                CCPDocumentInformation srcDocInfo = (CCPDocumentInformation) docInfoIter.next();
099                documentID = srcDocInfo.getDocumentID();
100            }
101            
102            if (documentID.equals("45023")) {
103                DEBUG = true;
104            } else {
105                DEBUG = false;
106            }
107            
108            /* This hash maps from the annotation Id to the annotation itself */
109            Map<Integer, CCPTextAnnotation> annotationIDMap = new HashMap<Integer, CCPTextAnnotation>();
110            /*
111             * This hash maps from the annotation ID to a flag indicating whether or not this annotation has been flagged for removal. Annotation removal
112             * occurs at the end of the process to avoid ConcurrentModificationExceptions.
113             */
114            Map<Integer, Boolean> annotationIDRemovedMap = new HashMap<Integer, Boolean>();
115    
116            int annotationID = 0;
117            Iterator textAnnotationIter = jcas.getJFSIndexRepository().getAnnotationIndex(CCPTextAnnotation.type).iterator();
118            while (textAnnotationIter.hasNext()) {
119                CCPTextAnnotation ccpTA = (CCPTextAnnotation) textAnnotationIter.next();
120    
121                boolean ignore = checkForIgnoreBasedOnAnnotationSet(ccpTA);
122    
123                if (!ignore) {
124                    if (ccpTA.getClassMention().getMentionName().equals(annotationTypeOfInterest)) {
125                        ccpTA.setAnnotationID(annotationID);
126                        annotationIDMap.put(annotationID, ccpTA);
127                        annotationIDRemovedMap.put(annotationID++, false);
128                    }
129                }
130            }
131    
132            List<CCPTextAnnotation> annotationsToRemove = new ArrayList<CCPTextAnnotation>();
133    
134            if (keepLongestCommonSubstring) {
135    //            keepLongestCommonSubstring(annotationIDMap, annotationIDRemovedMap, annotationsToRemove, jcas);
136                throw new UnsupportedOperationException("LongestCommonSubstring feature does not currently work.");
137            } else {
138                keepLongestAnnotation(annotationIDMap, annotationIDRemovedMap, annotationsToRemove, jcas);
139            }
140    
141            /* remove unnecessary annotations */
142            for (CCPTextAnnotation ccpTAtoRemove : annotationsToRemove) {
143                ccpTAtoRemove.removeFromIndexes();
144            }
145    
146            /* Remove duplicates */
147            annotationsToRemove = new ArrayList<CCPTextAnnotation>();
148            Set<String> spans = new HashSet<String>();
149            textAnnotationIter = jcas.getJFSIndexRepository().getAnnotationIndex(CCPTextAnnotation.type).iterator();
150            while (textAnnotationIter.hasNext()) {
151                CCPTextAnnotation ccpTA = (CCPTextAnnotation) textAnnotationIter.next();
152    
153                boolean ignore = checkForIgnoreBasedOnAnnotationSet(ccpTA);
154                if (!ignore) {
155                    if (ccpTA.getClassMention().getMentionName().equals(annotationTypeOfInterest)) {
156                        String spanStr = ccpTA.getBegin() + ".." + ccpTA.getEnd();
157                        if (spans.contains(spanStr)) {
158                            annotationsToRemove.add(ccpTA);
159                            if (DEBUG) {
160                                System.out.println("REMOVING DUPLICATE: ");
161                                UIMA_Util.printCCPTextAnnotation(ccpTA, System.out);
162                            }
163                        } else {
164                            spans.add(spanStr);
165                        }
166                    }
167                }
168            }
169            /* remove duplicate annotations */
170            for (CCPTextAnnotation ccpTAtoRemove : annotationsToRemove) {
171                ccpTAtoRemove.removeFromIndexes();
172            }
173        }
174    
175        /**
176         * keeps the longest annotation for a group of overlapping annotations
177         * 
178         * @param annotationIDMap
179         * @param annotationIDRemovedMap
180         * @param annotationsToRemove
181         * @param jcas
182         */
183        protected void keepLongestAnnotation(Map<Integer, CCPTextAnnotation> annotationIDMap, Map<Integer, Boolean> annotationIDRemovedMap,
184                List<CCPTextAnnotation> annotationsToRemove, JCas jcas) {
185            Set<Integer> idSet = annotationIDMap.keySet();
186            for (int id : idSet) {
187    
188                // while (textAnnotationIter.hasNext() & annotationsChecked < totalAnnotationsToCheck) {
189    
190                // annotationsChecked++;
191    
192                // CCPTextAnnotation ccpTA = null;
193                // try {
194                // ccpTA = (CCPTextAnnotation) textAnnotationIter.next();
195                // } catch (NoSuchElementException nsee) {
196                // /* If caught, then this annotation has already been removed from the CAS because it was overlapping another annotation */
197                // }
198                CCPTextAnnotation ccpTA = annotationIDMap.get(id);
199                if (ccpTA != null & !annotationIDRemovedMap.get(ccpTA.getAnnotationID())) {
200                    /*
201                     * assign an arbitrary annotation id so we can keep track of which annotations are identical when comparing overlapping annotations
202                     */
203                    // ccpTA.setAnnotationID(annotationsChecked);
204                    // System.err.println("AnnotationID: " + annotationsChecked);
205                    /* If it is a protein annotation, then look for overlapping annotations */
206                    // if (ccpTA.getClassMention().getMentionName().equals(ClassMentionTypes.PROTEIN)) {
207                    if (DEBUG) {
208                        System.err.println("CHECKING FOR OVERLAPS ON: ");
209                        UIMA_Util.printCCPTextAnnotation(ccpTA, System.err);
210                    }
211                    Span span;
212                    try {
213                        span = new Span(ccpTA.getBegin(), ccpTA.getEnd());
214                    } catch (InvalidSpanException e) {
215                        e.printStackTrace();
216                        span = null;
217                    }
218                    int longestAnnotation = ccpTA.getEnd() - ccpTA.getBegin();
219                    Iterator<CCPTextAnnotation> overlappingAnnotationsIter = UIMA_Util.getAnnotationsEncompassingSpan(span, jcas);
220                    debug(">0 overlapping annotations: " + overlappingAnnotationsIter.hasNext());
221                    /* Cycle through overlapping annotations, and remove if shorter than the original */
222    
223                    while (overlappingAnnotationsIter.hasNext()) {
224                        CCPTextAnnotation overlappingAnnotation = overlappingAnnotationsIter.next();
225                        if (DEBUG) {
226                            debug("Overlapping annotation: ");
227                            UIMA_Util.printCCPTextAnnotation(overlappingAnnotation, System.err);
228                        }
229    
230                        /* make sure the overlapping annotation is a protein */
231                        if (overlappingAnnotation.getClassMention().getMentionName().equals(annotationTypeOfInterest)) {
232    
233                            /* make sure we aren't supposed to ignore this annotation */
234                            boolean ignore = checkForIgnoreBasedOnAnnotationSet(overlappingAnnotation);
235                            if (!ignore) {
236                                /* make sure these are not the exact same annotation */
237                                if (ccpTA.getAnnotationID() != overlappingAnnotation.getAnnotationID()) {
238                                    int overlappingAnnotationLength = overlappingAnnotation.getEnd() - overlappingAnnotation.getBegin();
239                                    if (overlappingAnnotationLength <= longestAnnotation) {
240                                        if (DEBUG) {
241                                            System.err.println("THIS ANNOTATION IS SHORTER THAN THE LONGEST, SO WE WILL REMOVE IT: ");
242                                            UIMA_Util.printCCPTextAnnotation(overlappingAnnotation, System.err);
243                                        }
244                                        /* remove this annotation from the CAS */
245                                        annotationsToRemove.add(overlappingAnnotation);
246                                        annotationIDRemovedMap.remove(overlappingAnnotation.getAnnotationID());
247                                        annotationIDRemovedMap.put(overlappingAnnotation.getAnnotationID(), true);
248                                    } else {
249                                        if (DEBUG) {
250                                            System.err.println("THIS ANNOTATION IS LONGER THAN THE PREVIOUS LONGEST, SO WE WILL KEEP IT: ");
251                                            UIMA_Util.printCCPTextAnnotation(overlappingAnnotation, System.err);
252                                            System.err.println("AND THEN WE WILL REMOVE: ");
253                                            UIMA_Util.printCCPTextAnnotation(overlappingAnnotation, System.err);
254                                        }
255                                        /* keep this annotaition, and remove the previous longest */
256                                        annotationsToRemove.add(ccpTA);
257                                        annotationIDRemovedMap.remove(ccpTA.getAnnotationID());
258                                        annotationIDRemovedMap.put(ccpTA.getAnnotationID(), true);
259                                        longestAnnotation = overlappingAnnotationLength;
260                                    }
261                                }
262                            }
263                        }
264                    }
265                }
266            }
267        }
268    
269        /**
270         * keeps the longest common substring for a group of overlapping annotations
271         * 
272         * @param annotationIDMap
273         * @param annotationIDRemovedMap
274         * @param annotationsToRemove
275         * @param jcas
276         */
277        protected void keepLongestCommonSubstring(Map<Integer, CCPTextAnnotation> annotationIDMap,
278                Map<Integer, Boolean> annotationIDRemovedMap, List<CCPTextAnnotation> annotationsToRemove, JCas jcas) {
279            Set<Integer> idSet = annotationIDMap.keySet();
280            for (int id : idSet) {
281                CCPTextAnnotation ccpTA = annotationIDMap.get(id);
282                if (ccpTA != null & !annotationIDRemovedMap.get(ccpTA.getAnnotationID())) {
283                    if (DEBUG) {
284                        System.err.println("CHECKING FOR OVERLAPS ON: ");
285                        UIMA_Util.printCCPTextAnnotation(ccpTA, System.err);
286                    }
287                    Span span;
288                    try {
289                        span = new Span(ccpTA.getBegin(), ccpTA.getEnd());
290                    } catch (InvalidSpanException e) {
291                        e.printStackTrace();
292                        span = null;
293                    }
294                    // int longestAnnotation = ccpTA.getEnd() - ccpTA.getBegin();
295                    Iterator<CCPTextAnnotation> overlappingAnnotationsIter = UIMA_Util.getAnnotationsEncompassingSpan(span, jcas);
296                    debug(">0 overlapping annotations: " + overlappingAnnotationsIter.hasNext());
297                    /* Cycle through overlapping annotations, and remove if shorter than the original */
298    
299                    while (overlappingAnnotationsIter.hasNext()) {
300                        CCPTextAnnotation overlappingAnnotation = overlappingAnnotationsIter.next();
301                        if (DEBUG) {
302                            debug("Overlapping annotation: ");
303                            UIMA_Util.printCCPTextAnnotation(overlappingAnnotation, System.err);
304                        }
305    
306                        /* make sure the overlapping annotation is a protein */
307                        if (overlappingAnnotation.getClassMention().getMentionName().equals(annotationTypeOfInterest)) {
308    
309                            /* make sure we aren't supposed to ignore this annotation */
310                            boolean ignore = checkForIgnoreBasedOnAnnotationSet(overlappingAnnotation);
311                            if (!ignore) {
312                                /* make sure these are not the exact same annotation */
313                                if (ccpTA.getAnnotationID() != overlappingAnnotation.getAnnotationID()) {
314                                    /* now, alter the span of one annotation to equal the longest common substring, and then remove the other */
315                                    int newBegin = ccpTA.getBegin();
316                                    int newEnd = ccpTA.getEnd();
317                                    if (ccpTA.getBegin() < overlappingAnnotation.getBegin()) {
318                                        newBegin = overlappingAnnotation.getBegin();
319                                    }
320    
321                                    /* check to see if new substring annotation is valid */
322                                    if (newBegin >= newEnd | newBegin < 0 | newEnd < 1) {
323                                        System.err.println("INVALID SPAN!!!!!!!!!!!!!!!!!!!!!!       [" + newBegin + ".." + newEnd + "]");
324                                        UIMA_Util.printCCPTextAnnotation(ccpTA, System.err);
325                                        UIMA_Util.printCCPTextAnnotation(overlappingAnnotation, System.err);
326                                    }
327    
328                                    if (ccpTA.getEnd() > overlappingAnnotation.getEnd()) {
329                                        newEnd = overlappingAnnotation.getEnd();
330                                    }
331                                    try {
332                                        UIMA_Util.setCCPTextAnnotationSpan(ccpTA, newBegin, newEnd);
333                                    } catch (CASException e) {
334                                        e.printStackTrace();
335                                    }
336    
337                                    /* remove this annotation from the CAS */
338                                    annotationsToRemove.add(overlappingAnnotation);
339                                    annotationIDRemovedMap.remove(overlappingAnnotation.getAnnotationID());
340                                    annotationIDRemovedMap.put(overlappingAnnotation.getAnnotationID(), true);
341                                }
342                            }
343                        }
344                    }
345                }
346            }
347        }
348    
349        protected boolean checkForIgnoreBasedOnAnnotationSet(CCPTextAnnotation ccpTA) {
350            boolean ignore = false;
351            FSArray annotationSets = ccpTA.getAnnotationSets();
352            for (int i = 0; i < annotationSets.size(); i++) {
353                CCPAnnotationSet aSet = (CCPAnnotationSet) annotationSets.get(i);
354                if (annotationSetsToIgnore.contains(aSet.getAnnotationSetID())) {
355                    ignore = true;
356                    break;
357                }
358            }
359            return ignore;
360        }
361    
362        private void debug(String message) {
363            if (DEBUG) {
364                System.err.println("DEBUG -- OverlappingProteinResolver: " + message);
365            }
366        }
367    
368    }
```
